# Differences in Muscle Synergy Symmetry Between Subacute Post-stroke Patients With Bioelectrically-Controlled Exoskeleton Gait Training and Conventional Gait Training

**DOI:** 10.3389/fbioe.2020.00770

**Published:** 2020-07-29

**Authors:** Chun Kwang Tan, Hideki Kadone, Hiroki Watanabe, Aiki Marushima, Yasushi Hada, Masashi Yamazaki, Yoshiyuki Sankai, Akira Matsumura, Kenji Suzuki

**Affiliations:** ^1^Artificial Intelligence Laboratory, University of Tsukuba, Tsukuba, Japan; ^2^Faculty of Engineering, Information and Systems, University of Tsukuba, Tsukuba, Japan; ^3^Center for Innovative Medicine and Engineering, University of Tsukuba Hospital, Tsukuba, Japan; ^4^Center for Cybernics Research, University of Tsukuba, Tsukuba, Japan; ^5^Department of Neurosurgery, Faculty of Medicine, University of Tsukuba, Tsukuba, Japan; ^6^Department of Rehabilitation Medicine, Faculty of Medicine, University of Tsukuba, Tsukuba, Japan; ^7^Department of Orthopaedic Surgery, Faculty of Medicine, University of Tsukuba, Tsukuba, Japan

**Keywords:** muscle synergy, stroke, gait symmetry, robotic therapy, hybrid assistive limb (HAL^Ⓡ^)

## Abstract

Understanding the reorganization of the central nervous system after stroke is an important endeavor in order to design new therapies in gait training for stroke patients. Current clinical evaluation scores and gait velocity are insufficient to describe the state of the nervous system, and one aspect where this is lacking is in the quantification of gait symmetry. Previous studies have pointed out that spatiotemporal gait asymmetries are commonly observed in stroke patients with hemiparesis. Such asymmetries are known to cause long-term complications like joint pain and deformation. Recent studies also indicate that spatiotemporal measures showed that gait symmetry worsens after discharge from therapy. This study shows that muscle synergy analysis can be used to quantify gait symmetry and compliment clinical measures. Surface EMG was collected from lower limb muscles of subacute post-stroke patients (with an onset of around 14 days) from two groups, one undergoing robotic-assisted therapy (known as HAL group) and the other undergoing conventional therapy (known as Control group). Muscle synergies from the paretic and non-paretic limb were extracted with Non-Negative Matrix Factorization (NNMF) and compared with each other to obtain a gait symmetry index over therapy sessions. Gait events were tracked with motion tracking (for the HAL group) or foot pressure sensors (for the conventional therapy group). Patients from both groups were assessed over a 3-weeks long gait training program. Results indicated that there were no differences in muscle synergy symmetry for both groups of patients. However, the timing of muscle synergies were observed to be symmetrical in the HAL group, but not for the Control group. Intergroup comparisons of symmetry in muscle synergies and their timings were not significantly different. This could be due to a large variability in recovery in the Control group. Finally, stance time ratio was not observed to improve in both groups after their respective therapies. Interestingly, FIM and FMA scores of both groups were observed to improve after their respective therapies. Analysis of muscle coordination could reveal mechanisms of gait symmetry which could otherwise be difficult to observe with clinical scores.

## 1. Introduction

Gait impairment is traditionally associated with stroke, and hemiparesis is a common observance (Olney and Richards, 1996). As a result of weakness in one side of the body, gait asymmetries are notable features in the locomotion of such patients. Gait asymmetries are known to cause long-term complications, like inefficient energy expenditure, together with joint pain and deformation (Verma et al., 2012). Recently, studies indicated that gait asymmetries, like stance and swing symmetry are not adequately captured with conventional clinical measures, like gait velocity, motor deficit levels and impairment scores. Such clinical measures are uncorrelated with spatiotemporal measures of gait symmetry (e.g., step length, stance duration) (Patterson et al., 2010a; Rozanski et al., 2019). Although the earlier study (Patterson et al., 2010a) tracked patients up to 6 years post-stroke and reported that gait symmetry worsens, the more recent study by Rozanski et al. (2019) did not find the worsening of gait symmetry to be as severe. However, Rozanski et al. (2019) noted that since the monitoring was only performed for 6 months, they hypothesized that the possibility of gait symmetry worsening is high. They also pointed out that the number of patients who improved their gait symmetry after therapy was lower than expected, which is an indication that asymmetry of gait is difficult to correct (Rozanski et al., 2019).

Evidence of the neurological basis of gait symmetry can be observed in studies evaluating the symmetry of cortical connectivity in both hemispheres of the brain. Through the use of transcranial magnetic stimulation and magnetic resonance imaging, Madhavan et al. (2010) observed that patients with strong connectivity of the non-lesioned motor cortex to the paretic limb performed worse with the non-paretic ankle in a task to match a target with their ankle dorsiflexion and plantarflexion. Another similar study assessed side symmetry in the upper limbs by utilizing electroencephalogram and surface electromyography (EMG) (Graziadio et al., 2012). They provided evidence that neural activity in the non-lesioned side drives asymmetry and only measures of symmetry were correlated with global recovery scores (Graziadio et al., 2012). Taken together with clinical observations, there appear to be a correlation between gait symmetry and the symmetry of the nervous system, in terms of neural connections and strength of these connections. This suggests that improving gait symmetry could help improve this symmetry in connections. The rehabilitation approach of this study can be categorized as a “bottom-up” approach, where physical training or exercise is used as an intervention to influence the brain. Specifically in this study, we intend to evaluate the bottom-up effect of a biologically controlled exoskeleton which intervenes in the peripheral system, through which positive changes in the neural control of gait is expected. This is opposed to the “top-down” approach, where interventions are designed based on the state of the brain or to directly influence the brain with brain-computer interfaces (Belda-Lois et al., 2011). Further discussion on these two categories can be found in Belda-Lois et al. (2011).

Recently, exoskeletons have been developed for gait training and therapy for patients with neurological diseases (Jezernik et al., 2003; Hayashi et al., 2005; Veneman et al., 2007; Zeilig et al., 2012). These robots provide assistance to the lower limbs of patients for generating stepping motions in gait training. Studies evaluating the effects of such exoskeletons tend to focus on classic clinical outcomes, like gait velocity and functional recovery scores (Aach et al., 2014; Watanabe et al., 2014, 2017). However, despite the success of such exoskeletons, recent reviews noted that the benefits for therapy were still unclear and require further controlled studies to verify the effectiveness (Díaz et al., 2011; Louie and Eng, 2016). Therefore, there is a need to develop tools to understand the asymmetrical activity of the nervous system influencing gait recovery, as clinical evaluation scores are insufficient to provide insight about the state of the nervous system. In this case, muscle synergies could be one method worth considering as a clinical evaluation tool and for rehabilitation (Safavynia et al., 2011).

Muscle synergy analysis is a method that can be used to characterize muscle activation patterns in humans (Ivanenko et al., 2007; Torres-Oviedo and Ting, 2007). The hypothesis is that a small number of spatially grouped muscles (known as muscle synergies), and their corresponding timing coefficients, are sufficient to describe various locomotion tasks in gait and posture studies. This method also serves as a dimension reduction method for further analysis. Muscle synergies have been proposed as a manner the central nervous system reduces the complexity of controlling muscles to generate movement (Tresch and Jarc, 2009), and in recent years, have also been proposed to be related to motor primitives (Giszter, 2015).

As for its applications, muscle synergies has also been shown to be robust between subjects (Chvatal and Ting, 2012) and even between days (Shuman et al., 2016). Muscle synergy analysis has also been successfully applied on assessing gait performance in stroke patients (Clark et al., 2010; Gizzi et al., 2011; Routson et al., 2013; Barroso et al., 2017). Hence, to allow better characterization of gait symmetry change, the use of muscle synergy analysis is proposed to analyze muscle coordination changes that occur over the course of different types of therapy, specifically in this study, the difference between robotic-assisted and conventional gait training.

A related study (Patterson et al., 2015), evaluated changes in spatiotemporal gait asymmetry during in-patient rehabilitation. This study was motivated by the lack of information about how patients change their spatiotemporal gait symmetry over the course of therapy. Their main findings was that a majority of patients did not significantly improve their gait symmetry during the course of therapy and after discharge. The use of muscle synergy analysis would be beneficial in such situations, where spatiotemporal gait measures are unable to differentiate changes in gait of stroke patients over therapy. A previous study (Tan et al., 2018) showed that a course of robotic therapy with a bioelectrically-controlled exoskeleton was effective in restoring gait symmetry, as quantified by muscle synergies. However, that study was limited to accessing the outcome of patients after robotic therapy, and no comparison was performed with patients that did not undergo robotic therapy. Another similar study also used muscle synergy analysis to examine differences between lower limbs of spinal cord injury patients (Pérez-Nombela et al., 2017). They found that there were differences in the composition and activation of muscle synergies between lower limbs, suggesting that spinal cord injury patients suffer from a similar problem in stroke patients, where one limb is more affected that the other limb.

This current study aims to address the limitations of the previous study by evaluating the short-term changes in spatial and temporal muscle coordination symmetry, as quantified by the spatial organization of muscles used (muscle synergies) with their corresponding activation times (timing coefficients), in patients undergoing a course of robotic-assisted gait training and compare them with patients undergoing a course of conventional gait training.

## 2. Methods

To evaluate the effects of robotic gait therapy on muscle coordination symmetry, subacute post-stroke patients were recruited and divided into two groups, with one group undergoing robotic gait training, while the other group underwent conventional gait training. Muscle coordination differences between groups were evaluated before, after and during the course of therapy. Clinical test scores, stance duration and stance time ratio changes were also reported.

### 2.1. Participants

This study was carried out in accordance with the recommendations of the University Guidelines for Clinical Trials, Institutional Review Board of University of Tsukuba Hospital, with written informed consent from all subjects. All subjects gave written informed consent in accordance with the Declaration of Helsinki. The protocol was approved by the Institutional Review Board of University of Tsukuba Hospital.

Patients were recruited in a decentralized manner from the University of Tsukuba Hospital, Ibaraki Kennan Hospital, Kobari Sogo Clinic, Tsukuba Memorial Hospital, and the Ibaraki Seinan Iryo Center Hospital. They were assigned without randomization based on the hospitals they were admitted to.

Patients recruited from the University of Tsukuba Hospital were assigned to the robotic gait therapy group (known as HAL group thereafter), while patients from the other hospitals were assigned to the conventional therapy group (known as Control group thereafter). Patients exhibiting hemiparesis after unilateral ischemic or hemorrhagic stroke, aged between 40 and 80, were examined by the Functional Ambulation Categories (FAC) criteria for inclusion (FAC score of either 1 or 2). Patients who had consciousness issues, cardiac disease (defined as myocardial infarction, severe heart failure, arrhythmia, or cardiomyopathy presenting abnormal blood pressure, heart rate or SpO2) or musculoskeletal problems were excluded. All patients arriving in the participating hospitals due to acute stroke were examined by the above criteria and recruited into the study if they fulfill the conditions. Numbers of patients recorded were only for those that fulfilled the criteria. Due to the difficulty in recruiting patients and matching intervention schedules between the groups across different hospitals, sample sizes were determined based on convenience, where at least 10 patients per group was set to be the target size.

Data of patients in the HAL group from the previous study (Tan et al., 2018) (Table 1 R1–R8) were used for analysis in this current study. Data of new patients (Table 1 R9–R11) that recently completed their course of therapy were included as well, making a total of four males and seven females patients. HAL group patients were aged between 43 and 80 (60.3 ± 11) years old. They were included in the study about 10–18 (13.9 ± 3.2) days after the onset of stroke.

**Table 1:** Participants characteristics.

**ID**	**Age (years)**	**Gender**	**Diagnosis**	**Affected (side)**	**Onset- eval for eligibility (days)**	**Onset-1st session (days)**	**FAC at recruit**	**FAC at 1st session**
R1	67	F	CI	L	8	10	1	1
R2	52	F	ICH	R	13	17	1	2
R3	71	F	CI	L	7	11	1	1
R4	55	M	CI	L	8	10	2	2
R5	55	F	CI	L	14	16	2	3
R6	43	M	CI	R	8	11	1	2
R7	51	F	CI	R	15	18	2	2
R8	80	M	CI	R	14	16	2	2
R9	61	F	ICH	L	8	12	2	3
R10	72	F	ICH	R	12	14	1	1
R11	56	M	ICH	R	15	18	1	1
Mean ± SD	60.3 ± 13.9				11.1 ± 3.3	13.9 ± 3.2	1.5 ± 0.5	1.8 ± 0.8
C1	76	M	ICH	R	15	17	1	1
C2	69	F	ICH	L	9	14	1	2
C3	64	M	ICH	L	14	15	1	1
C4	49	M	ICH	R	16	18	1	2
C5	69	F	CI	L	10	17	1	2
C6	66	F	CI	L	14	12	2	2
C7	73	M	ICH	R	10	16	2	2
C8	65	M	CI	R	15	18	2	2
C9	53	M	CI	L	15	14	2	2
Mean ± SD	64.9 ± 8.8				13.1 ± 2.7	15.7 ± 2.1	1.4 ± 0.5	1.8 ± 0.4

*Diagnosis was classified into Cerebral Infarction (CI) and Intracerebral Hemorrhage (ICH). HAL patients were labeled with the “R” prefix in their IDs (R1–R11), while conventional therapy patients were given the “C” prefix (C1–C9). Note that there is a difference of a few days between the evaluation for study eligibility and start of actual gait training*.

Initially, the Control group comprised of seven males and four females subacute stroke patients. However, two patients dropped out of the study in the first session, citing discomfort with removing their clothing for the attachment of EMG electrodes, especially for the gluteus maximus electrode. Subjects that dropped out continued with their therapies at their respective hospitals, but no additional data was collected from them as they left the study. The remaining six males and three females stroke patients (Table 1 C1–C9) underwent conventional gait training, with training schedules matched to the HAL group. Patients were aged between 49 and 76 (64.9 ± 8.9) years old. The control group were included in the study about 12–18 (15.7 ± 2.1) days after the onset of stroke.

Robotic gait training and all evaluations for the HAL group were performed in the University of Tsukuba Hospital, while conventional gait training and all evaluations for the Control group were performed at the following hospitals and clinics : Ibaraki Kennan Hospital, Kobari Sogo Clinic, Tsukuba Memorial Hospital, Ibaraki Seinan Iryo Center Hospital. Attachment of sensors and operation of the measurement equipment were performed by the same staff member who performed data capture for the HAL group. The staff member traveled to participating hospitals and clinics during the measurement of the patients in the Control group.

### 2.2. Gait Training Methods

In addition to gait training described here, both groups of patients (HAL group and Control group) received a total of 160 min per week of conventional regular physiotherapy as part of their rehabilitation during their subacute phase, in their respective hospitals.

#### 2.2.1. HAL Group

The single leg version of Robot Suit HAL (Hybrid Assistive Limb) (Hayashi et al., 2005) was used for patients in the HAL group on their paretic limb. The robot was composed of four rigid segments (lumbar, thigh, shank and shoe), actuated with motors in the hip and knee joints. The robot is able to function in two modes, the CVC (Cybernic Voluntary Control) and CAC (Cybernic Autonomous Control) modes. Details of the control modes are as follows:

- **CVC mode:** EMG signals were detected from the surface of the skin over the hip flexor (Illiopsoas) and extensor muscles (Gluteus Maximus), as well as, the knee flexors (Hamstring) and extensor muscles (Vastus Lateralis). The ratio between the flexor and extensor muscles determines the direction and amount of assistive torque that is to be generated in real time. Gain parameters can be set individually for each flexor or extensor muscle by the therapist until the patient is comfortable with controlling the robot.
- **CAC mode:** Assistance is generated based on a reference gait pattern from healthy subjects. The robot generates a pre-planned joint trajectory according to the gait phase detected by the joint angle and foot pressure sensors embedded in the shoe segment of the robot.

Patients followed the protocol detailed in Tan et al. (2018). Briefly, HAL therapy was started during the participants' subacute period (Table 1). For each patient in the HAL group, overground gait training were performed three times per week for 3 weeks (9 sessions), with the exoskeleton. Each training session lasted for 20 min, where patients walked in a 25 m course, composed of two straight lines and two semicircles. Breaks were provided as needed. No specific instructions were provided to the patients, other than the encouragement to walk, since the robot exoskeleton intervenes by providing assistance based on the remaining EMG signals from their lower limb muscles or the gait phase, depending on the control mode used. For safety and fall prevention, a walking device (All-in-One Walking Trainer, Ropox A/S, Naestved, Denmark) with a harness was used, but no body weight support was provided. Only 1 patient in this group started the program with CAC and progressed to CVC. The rest of the patients were able to utilize the CVC mode from the beginning of the program.

#### 2.2.2. Control Group

For each patient in the Control group, the same amount of overground gait training as the HAL group was performed (three sessions each week for a total of nine sessions). Each training session for patients in this group also lasted for 20 min and breaks were provided as needed.

### 2.3. Data Measurement

#### 2.3.1. Data Collection Protocol

Lower limb movement of patients in the HAL group was measured with a motion capture system (detailed in section 2.3.3). Lower limb muscle activity were measured with wireless EMG electrodes (detailed in section 2.3.2). Measurement was conducted during straight-line walking, at a self-selected speed without wearing HAL. Measurement schedule are as follows: before the 1st session, before the 4th session, before the 7th session, and after the 9th session. The All-In-One Walking Trainer (Ropox A/S, Denmark), with a harness, was used during the walking test to prevent falls. The harness was adjusted such that it did not provide any weight support. The patients walked for 6 m several times in order to maximize the number of strides for collection. Also, the initiation and termination of walking during each 6 m walking trial were discarded as well.

Gait of patients in the conventional gait training group was measured with the same protocol as the HAL group (self-selected walking speed, 6m walking distance, All-in-One Walking training with harness for fall prevention, harness did not provide weight support, and 6m walking test was conducted several times to maximize the number of gait cycles collected). Measurement schedule was matched with the HAL group (before course of therapy, before 4th session, before 7th session, after 9th session). Lower limb muscle activity were measured with the same EMG system defined in section 2.3.2. However, due to the lack of a motion tracking system for this group, gait events (heel strike and toe off) were determined with foot pressure sensors, detailed in section 2.3.4.

#### 2.3.2. Electromyography (EMG)

Skin preparation included wiping down the muscle bellies with alcohol swabs. Twelve wireless, surface EMG electrodes were placed bilaterally over the muscle bellies of Vastus Medialis (VM), Hamstrings [Semitendinosus] (HAM), Tibialis Anterior (TA), Gastrocnemius [Medial Head] (GAS), Adductor Longus (ADD), Gluteus Maximus (Gmax), using a Trigno^*TM*^ Lab Wireless EMG system (Delsys Inc., Boston, MA, USA). EMG data was sampled at 2,000 Hz. This data measurement protocol was applied on both groups of patients.

#### 2.3.3. Motion Tracking

For the HAL group, motion tracking of subjects was achieved with a motion capture system (VICON MX System with 16 T20S Cameras, Vicon, Oxford, UK), in synchronization with EMG and sampled at 100 Hz. Sixteen autoreflective markers were placed bilaterally on the anterior superior iliac spine, posterior superior iliac spine, lower lateral 1/3 surface of the thigh, flexion-extension axis of the knee, lower lateral 1/3 surface of shank, lateral malleolus for the ankle, posterior peak of the calcaneus for the heel and the lateral second metatarsal bone of the toe. These marker positions were used to determine gait phase during locomotion.

#### 2.3.4. Foot Pressure Sensor

For the Control group, gait phase was determined with foot pressure sensors, Trigno^*TM*^ 4-channel FSR (Force Sensitive Resistor) (Delsys Inc., Boston, MA, USA), sampled at 100 Hz. Two FSRs were used, with a FSR pasted below the big toe and the other pasted below the heel of patients. Shoes from the same manufacturer were provided for the patients to ensure that FSR values were not affected by different shoe types. Gait phase detection was based on the pressure sensor values.

#### 2.3.5. Verification Between Vicon and Foot Pressure Sensors

A small verification test was conducted to check the differences in measurement values between the motion tracking system and foot pressure sensors. Data from 3 healthy subjects were collected for overground walking. Similar to the Control group, foot pressure sensors (Delsys, Trigno^*TM*^ 4-channel FSR (Force Sensitive Resistor), sampled at 100 Hz) were used, with 1 FSR pasted below the big toe and the other pasted below the heel. Shoes, which have the same manufacturer as the Control group, were provided. The same motion capture system (VICON MX System with 16 T20S Cameras, Vicon, Oxford, UK, sampled at 100 Hz), was also used. 6 reflective markers were placed bilaterally on the lateral malleolus for the ankle, posterior peak of the calcaneus for the heel, and the lateral second metatarsal bone of the toe. Subjects walked for five trials of 10 m each, at a self-selected speed. Heel-strike and toe-off events were recorded for both legs in order to calculate stance duration for both legs. The absolute error between the values from both measurement systems were calculated.

### 2.4. Clinical Assessments

Clinical evaluation were conducted at the 1st session and after the 9th session with the Functional Independence Measure (FIM) and Fugl-Meyer Assessment (FMA) as listed below:

1. FIM—Locomotion
2. FIM—Motor (General)
3. FMA—LE (Lower Extremity)

The temporal gait parameter, stance duration, was captured as it has been shown to be a relatively good indication of symmetry in other studies (Patterson et al., 2010a). The measure used here is the stance duration ratio, which was defined in Patterson et al. (2010b) as:

(1) $$\begin{array}{r} \text{stance ratio}=T_{paretic}/T_{non-paretic} \end{array}$$

where *T*_*paretic*_ and *T*_*non*−*paretic*_ are the stance duration of both the paretic and non-paretic side, respectively, expressed in percentages of the gait cycle.

### 2.5. Data Analysis

#### 2.5.1. Pre-processing

The extracted EMG data was first band-passed with a 4th order, zero-lag Butterworth filter at 30–400 Hz. The bandpassed EMG was then filtered with a Hampel filter (parameters : time window—win = 200, threshold—σ = 4) to remove artifacts in EMG data. Finally, the EMG data was fully rectified and low-passed at 6 Hz, with a 4th order, zero-lag Butterworth filter.

#### 2.5.2. Extraction of Gait Events

For the HAL group the elevation of the heel markers were used to identify gait events. A heel strike is determined to be the point where elevation of the heel reflective marker is at the lowest point. A toe-off is determined to be at the point right before a steep increase in elevation of the toe reflective marker.

From all the gait events collected, gait cycles (strides) from each lower limb were extracted from the gait event recordings of each trial. Data indices between two consecutive heel strikes were considered as a stride. Strides were separated into “Paretic” and “Non-Paretic” categories, based on the paretic side of the patients as assessed by medical personnel. A selection criteria was imposed to select consistent gait cycles. This criterion is to filter out steps where patients stumble, which is a common occurrence during the early stages of the course of therapy (1st and 4th session). The selection criteria is as follows:

- Stride times for each lower limb, per session, was calculated from the indices of heel strikes (Paretic stride time, Non-Paretic stride time). Stride times were combined from multiple walk tests.
- A histogram of stride times were calculated for both the paretic limb and non-paretic limb.
- The bin width was determined with the Freedman-Diaconis rule. This was achieved with Matlab's histcount function.
- The strides that belong to the bin with the highest count were selected for further analysis.

**Figure 1:**
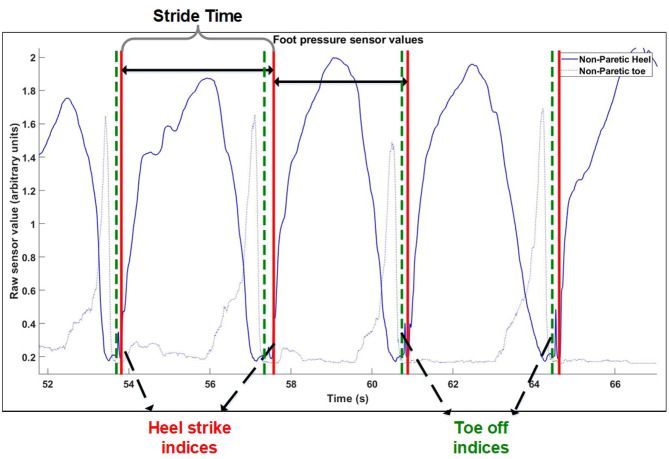
Gait cycle extraction method. Extraction of windows of consecutive steps for control group.

The process for extraction of gait events was the similar for the Control group, except that instead of motion tracking, foot pressure sensors were used. The process of extracting heel strikes from the control group is illustrated in Figure 1. A heel strike is determined to be the start of the rising edge of heel pressure sensor values, while a toe off is determined to be the end of the falling edge of the toe pressure sensor value (Figure 1).

#### 2.5.3. Extraction of EMG

Preprocessed EMG data (section 2.5.1) of selected strides were separated into Paretic and Non-paretic windows (Paretic side, Non-paretic side), using the best heel strike indices obtained from section 2.5.2. EMG envelopes from each gait cycle was then normalized by dividing each EMG channel with its standard deviation, following the definition of “UnitPer” described in Banks et al. (2017). The normalized EMG envelopes of each stride were then interpolated to 100 time points and concatenated together (Oliveira et al., 2014), giving a matrix of 6 by (100·N) (6 EMG channels of 100 time points multiplied by the number of strides selected by the selection criteria), for each lower limb.

#### 2.5.4. Muscle Synergy Extraction With NNMF

Non-negative Matrix Factorization (NNMF) (Lee and Seung, 1999) was used to extract muscle synergies from concatenated EMG data. This was performed with Matlab's NNMF function, using the multiplicative update algorithm. Parameters for the tolerance for the residual (TolFun) was set to 10^−6^ and the tolerance for the relative change in elements (TolX) was set to 10^−4^. The algorithm was repeated 300 times and results with the lowest root mean square residual were taken to be the best. Synergies were allowed to vary per condition.

The choice of number of synergies was determined with the criteria of when the overall variance-accounted-for (VAF_*total*_) between the reconstructed and original EMG envelope was above 90%. A local criteria imposed was that the reconstruction VAF for each muscle (VAF_*muscle*_) was above 75% and that and subsequent increase of the number of synergies did not give more than a 5% increase in the mean VAF of all muscle channels. The VAF is defined as 100*(uncentered Pearson correlation coefficient), which requires the total sum of squares to be taken with respect to zero (Torres-Oviedo and Ting, 2007). This is given as:

(2) $$\begin{array}{r} VAF=100\cdot \left(\frac{{\left(\overset{m}{\underset{j=1}{\sum }}\overset{n}{\underset{i=1}{\sum }}X_{nm}\cdot Y_{nm}\right)}^{2}}{\left(\overset{m}{\underset{j=1}{\sum }}\overset{n}{\underset{i=1}{\sum }}X_{nm}^{2}\right)\cdot \left(\overset{m}{\underset{j=1}{\sum }}\overset{n}{\underset{i=1}{\sum }}Y_{nm}^{2}\right)}\right) \end{array}$$

where *n* is the number of data points for each channel, and *m* is the number of channels. *X*_*nm*_and*Y*_*nm*_ are the matrices containing the reconstructed and original signal, respectively.

#### 2.5.5. Synergy Analysis

**Figure 2:**
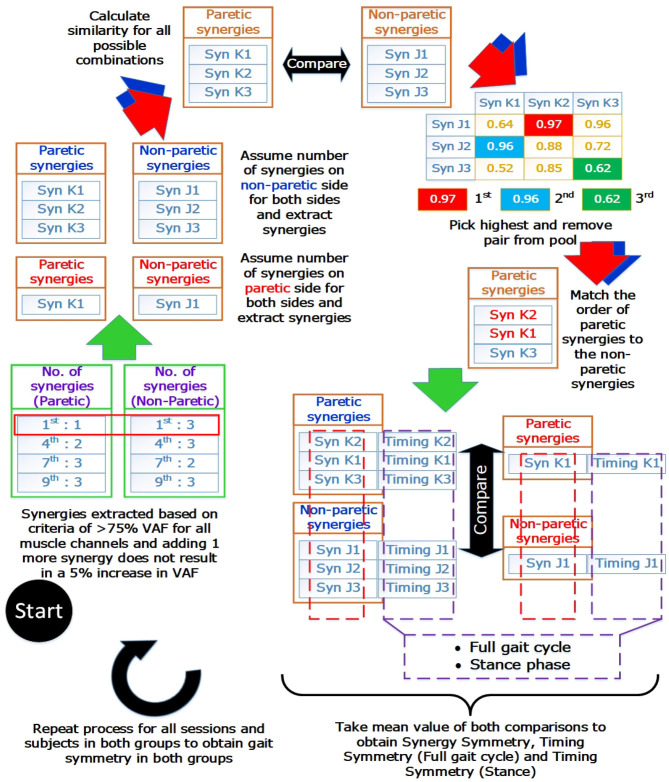
Muscle synergy matching Infograph. Graphical representation of matching muscle synergies on the paretic side to the non-paretic side. Similarity between each synergy is quantified with the scalar dot product(or known as cosine similarity) and similarity between timing coefficients is quantified with the Pearson correlation coefficient. Timing coefficients used for full gait cycle calculations were extracted from the concatenated results and averaged, giving 100 time points for comparison. Timing coefficients used for stance phase was extracted with the stance percent, interpolated to 60 time points and averaged before comparison.

Prior to comparison, muscle synergies on the paretic side were matched according to the muscle synergies on the non-paretic side. The similarities of muscle synergies on the paretic side to the non-paretic side were calculated with the scalar dot product (Cheung et al., 2012). The pair with the highest similarity score was removed from the pool and the process continues until all muscle synergies were matched. This matching process was repeated for all sessions and subjects. After the matching process, synergies and timing coefficients were compared to obtain a value to denote the symmetry between them. These values will be referred to as the “synergy symmetry” (for synergy weight symmetry) and “timing symmetry” (for timing coefficient symmetry). An infograph of the matching process is provided in Figure 2.

Typically, the number of synergies were chosen based on a threshold value of the VAF (Torres-Oviedo and Ting, 2007; Clark et al., 2010). However, this would mean the paretic and non-paretic side will have different number of synergies, with the paretic side usually having a smaller number of synergies due to merging of synergies (Cheung et al., 2012). This makes direct comparison between the synergies difficult. Hence, by imposing the same number of synergies on both the paretic and non-paretic side, direct comparison becomes possible. However, to prevent information loss with such a method, all possible number of synergies will have to be considered during analysis. From the example shown in Figure 2 (Blue arrows and synergies in blue), synergies were matched by assuming that the same number of synergies were present on the paretic side, using the number of synergies from the non-paretic side (Figure 2, “Assume number of synergies on *non-paretic side* for both sides”). This process was then repeated until all synergies for all possible conditions and sessions were matched. Labels for the results section will be shortened using the labels shown below:

- *Assume*_*Non*−*paretic*_: Assume number of synergies on *non-paretic side* for both sides
- *Assume*_*Paretic*_: Assume number of synergies on *paretic side* for both sides.

After the matching process, synergies on both sides of the body were compared with the scalar dot product and the mean of each comparison was recorded. Additionally, the similarity of the corresponding timing coefficients for the muscle synergies were evaluated with the Pearson correlation coefficient, R. Evaluation was done with the mean of the timing coefficients of 3 steps. This is to account for step-to-step variability.

#### 2.5.6. Software

Data extraction from the Motion capture and EMG systems was done using custom scripts on MATLAB 8.4 (Mathworks, Natick, MA, USA). NNMF and the rest of the processing were performed with custom scripts on MATLAB 9.3 (Mathworks, Natick, MA, USA). Statistical tests were performed with custom scripts on R (version 3.5.3).

### 2.6. Statistical Analysis

Statistical analysis of the data was performed with the Paired Wilcoxon Signed-rank Test for comparison of clinical scores, muscle synergy symmetry and stance duration within groups. Due to unequal group sizes, intergroup comparisons of muscle synergy symmetry and stance duration were evaluated with the Mann-Whitney *U*-Test. Significance was considered in comparisons with *p* < 0.05 with 95% confidence intervals (CI) reported. Statistical analysis was performed with non-parametric tests as normality of the distribution cannot be assumed.

A preliminary two-way ANOVA was used on the obtained symmetry values to check for interaction between the choice of number of synergies with muscle synergy and timing symmetry values (pre-therapy or post-therapy). This is to check if selecting different number of synergies would cause gait symmetry to be estimated differently. There was no significant interaction between the different choices of number of synergies and muscle synergy symmetry (*p* = 0.6079), timing symmetry for the full gait cycle (*p* = 0.3079), and timing symmetry for the stance phase (*p* = 0.3688). This indicates that there is no interaction between choosing different number of synergies and symmetry values.

## 3. Results

Patients labeled R5 and R9 were excluded from analysis as their FAC values during the 1st session were at 3. This is to ensure that the inclusion criteria was adhered to during analysis. However, since the patients participated in the study, results of these two patients were presented individually.

### 3.1. Patient Characteristics

The age of patients between groups did not significantly differ [HAL group (60.78 ± 12.16) vs. Control group (64.88 ± 8.79)] (*p* = 0.5961, *CI* = [−17.0000, 7.0000]). The duration from the onset of stroke to the first session of gait training did not differ as well [HAL group (13.9 ± 3.4) vs. Control group (15.7 ± 2.1)] (*p* = 0.3046, *CI* = [−6.0000, 2.0000]) (Table 1). Group comparisons of FIM-Locomotion, FIM-Motor, and FMA-LE scores at the 1st session were only significantly different for FIM-locomotion, but not for FIM-Motor and FMA-LE (FIM-Locomotion: *p* = 0.0395, *CI* = [−2.0001, 0.0000]) (FIM-Motor: *p* = 0.8944, *CI* = [−13.0000, 16.0000]) (FMA-LE: *p* = 0.9295, *CI* = [−9.9999, 10.0000]) (Table 2).

**Table 2:** Clinical evaluation scores at the 1st session (Pre) and after the 9th session (Post).

**ID**	**FIM- Locomotion (Pre)**	**FIM- Locomotion (Post)**	**FIM-Motor (General) (Pre)**	**FIM-Motor (General) (Post)**	**FMA-LE (Pre)**	**FMA-LE (Post)**
R1	1	3	46	73	13	18
R2	1	5	40	82	19	26
R3	1	2	40	55	18	28
R4	2	7	52	77	26	29
R6	1	6	66	83	21	25
R7	1	1	53	62	14	22
R8	1	5	50	65	17	20
R10	1	5	62	83	26	30
R11	1	1	60	72	14	20
Mean ± SD	1.1 ± 0.3	3.9 ± 2.2	52.1 ± 9.3	72.4 ± 10	18.7 ± 4.9	24.2 ± 4.4
C1	2	3	29	35	3	10
C2	3	5	55	64	12	24
C3	1	2	18	48	9	16
C4	1	1	54	76	24	25
C5	1	1	46	64	9	18
C6	5	6	62	86	27	33
C7	3	5	67	71	29	33
C8	3	5	65	83	25	27
C9	1	6	50	87	25	34
Mean ± SD	2.2 ± 1.4	3.8 ± 2.0	49.5 ± 16.5	68.2 ± 17.7	18.1 ± 9.7	24.4 ± 8.4

*Patients with the “R” prefix belong to the HAL group, while patients with the “C” prefix belong to the Control group*.

### 3.2. Clinical Scores

The FIM-Locomotion score (*p* = 0.0213, *CI* = [−4.5000, −2.5000]), FIM-Motor (General) score (*p* = 0.0091, *CI* = [−28.5000, −12.9999]), FMA-LE scores (*p* = 0.0090, *CI* = [−7.5000, −3.5000]) increased in the HAL group (Table 2 R1–R11). Patients in the Control group (Table 2 C1–C9) had significantly increased clinical scores in all categories, pre- and post-therapy [FIM-locomotion (*p* = 0.0206, *CI* = [−3.5000, −1.0000]), FIM-Motor (General) (*p* = 0.0091, *CI* = [−27.5001, −9.0000]) and FMA-LE (*p* = 0.0090, *CI* = [−9.0001, −3.4999])].

### 3.3. Overview of EMG

**Figure 3:**
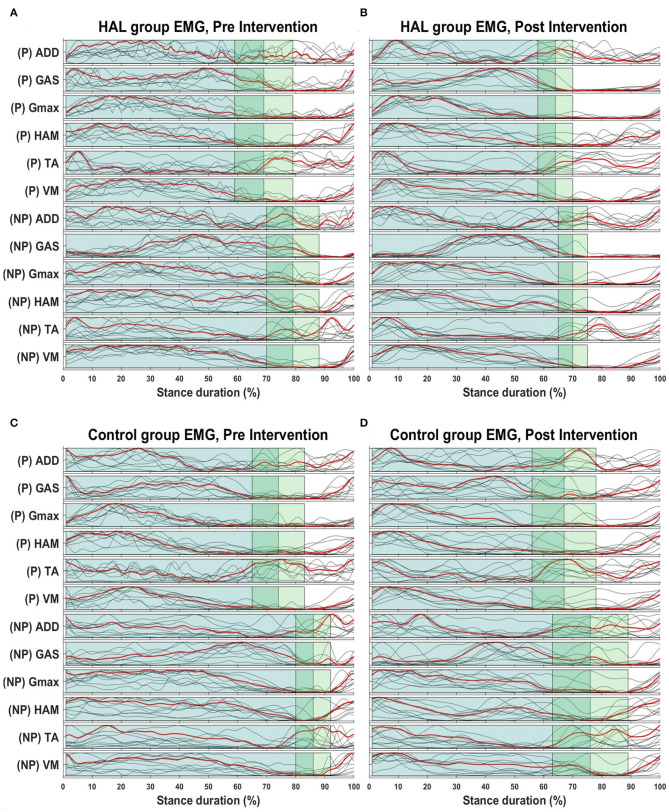
Overview of EMG envelopes and stance percentage. Overview of changes in stance percentage and EMG waveform for both HAL **(A,B)** and Control group **(C,D)**. Dark green shaded areas represent the mean stance percentage for all patients in their respective groups, while the lighter green areas represent the standard deviation. Red lines indicate the mean EMG amplitudes for the all patients in their respective groups, while the gray lines represents mean EMG waveform from each patient.

Figure 3 below provides a graphical overview relating the change in the EMG and stance duration in percentage. The first two subfigures (Figures 3A,B) illustrate the mean changes in the HAL group, while the following two (Figures 3C,D) illustrate mean changes in the Control group.

### 3.4. Stance Duration and Stance Time Ratio

**Figure 4:**
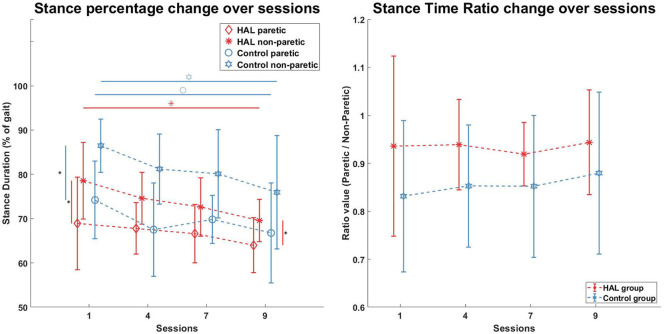
Stance percentage and Stance Ratio. Stance duration in percentages was significantly lower for the Non-Paretic limb in the HAL group after therapy (Red horizontal line, Red asterisk), but not in the Paretic limb. Stance duration in both limbs of the Control group were significantly lower after therapy (Blue horizontal lines, blue circle, and star). Stance asymmetry in both groups were present at the beginning (Red vertical line, blue vertical line, 1st session) but only the Control group became more symmetric after therapy, while the HAL group remains asymmetric (Red vertical line, 9th session). Stance Time ratios were not significantly different within groups and between groups.

Stance duration, expressed as a percentage of the gait cycle (heel strike to heel strike), was evaluated and shown in Figure 4 (Left). A significant decrease in stance duration was observed in the HAL group for the non-paretic limb after therapy [78.6 ± 8.7% -> 69.6 ± 4.8% (*p* = 0.0078, *CI* = [0.0353, 0.1619])], marked with an asterisk in Figure 4 (Left—Red horizontal line with asterisk). However, the stance duration of the paretic leg was not significantly decreased [68.9 ± 10.5% -> 64.0± 6.2% (*p* = 0.1289, *CI* = [−0.0745, 0.1528]) (Paretic leg)]. For the Control group, a significant decrease in stance duration was observed in the both legs [74.2 ± 8.8% -> 66.8 ± 11.3% (*p* = 0.0391, *CI* = [0.0009, 0.1454]) (Paretic), [86.5 ± 6% -> 75.9 ± 12.8% (*p* = 0.0078, *CI* = [0.0396, 0.2088]) (Non-Paretic)], Figure 4 (Left—Blue horizontal lines with circle and star symbols)]. Marginal significant differences was observed for non-paretic stance duration between the HAL group and Control group in the 1st session (*p* = 0.0503, *CI* = [−0.1880, 0.0023]) [indicated with a vertical line and diamond in Figure 4 (Left)], but differences were significant in the 9th session (*p* = 0.0315, *CI* = [−0.1995, −0.0021]) (indicated with a vertical line and asterisk in Figure 4 Left). However, no significant differences were observed in the paretic stance duration between groups before and after their respective therapies.

Stance time ratio for both groups were not significantly different before and after the course of therapy for both groups. Statistical comparison of the stance time ratio between both groups were also not significant.

### 3.5. Number of Muscle Synergies

The number of synergies that are able to fulfill the VAF criteria (>90% VAF overall, >75% VAF per muscle channel and increase in 1 number of synergy does not results in a 5% increase in mean VAF from every muscle channel), are listed in Table 3. Changes in the number of synergies after the 9th session, for both the paretic and non-paretic limbs, are listed in brackets. Five patients in the HAL group had an increase in the number of synergies in the paretic limb (R1, R2, R6, R8, R11), as compared to 3 in the Control group (C1, C3, C7). For the non-paretic limb, three patients in the HAL group showed changes (R4, R7, R11), while it was four patients in the Control group (C1, C3, C7, C8). More patients in the HAL group showed no difference in the number of synergies between the paretic and non-paretic limbs after the 9th session, as compared to the Control group (5 in HAL group against 1 in Control group).

**Table 3:** Table listing number of synergies in limbs of patients.

**ID**	**Paretic limb**		**Non-paretic limb**		**Difference between limbs**	
	**1st session**	**9th session**	**1st session**	**9th session**	**1st session**	**9th session**
R1	1	3 (+2)	3	3 (+0)	2	0
R2	2	4 (+2)	3	3 (+0)	1	1
R3	2	2 (+0)	3	3 (+0)	1	1
R4	3	3 (+0)	4	3 (−1)	1	0
R6	2	3 (+1)	3	3 (+0)	1	0
R7	1	1 (+0)	2	3 (+1)	1	2
R8	2	3 (+1)	3	3 (+0)	1	0
R10	4	2 (−2)	2	2 (+0)	2	0
R11	2	3 (+1)	3	2 (−1)	1	1
C1	2	3 (+1)	2	4 (+2)	0	1
C2	2	2 (+0)	3	3 (+0)	1	1
C3	1	2 (+1)	4	3 (−1)	3	1
C4	2	2 (+0)	3	3 (+0)	1	1
C5	1	1 (+0)	3	3 (+0)	2	2
C6	1	1 (+0)	2	2 (+0)	1	1
C7	2	3 (+1)	3	2 (−1)	1	1
C8	3	3 (+0)	4	3 (−1)	1	0
C9	3	3 (+0)	4	4 (+0)	1	1

*Changes in the number of synergies in the same limb after therapy are listed in brackets*.

### 3.6. Muscle Synergy Symmetry

**Figure 5:**
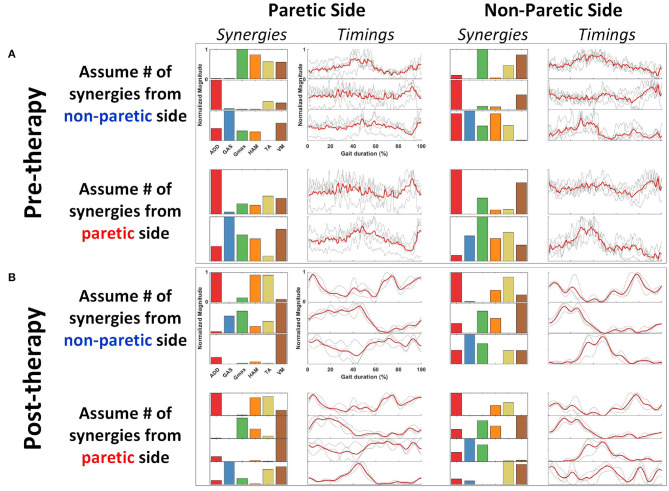
Representative subject (R2) with all synergy extraction parameters. Figures are arranged as **(A)** Pre-therapy, Paretic Side (Left column), Non-Paretic Side (Right column). **(B)** Post-therapy, Paretic Side (Left column), Non-Paretic Side (Right column). Rows for both pre- and post-therapy conditions show the synergies extracted with the assumptions in number of synergies. Synergies and timing coefficients are scaled to have values between 0 and 1.

The figure below provides an example how would muscle synergies extracted with the comparison conditions described in section 2.5.5 look like (Figure 5). A representative subject, R2, was selected from the HAL group because the patient has the most number of muscle synergy change throughout therapy.

**Figure 6:**
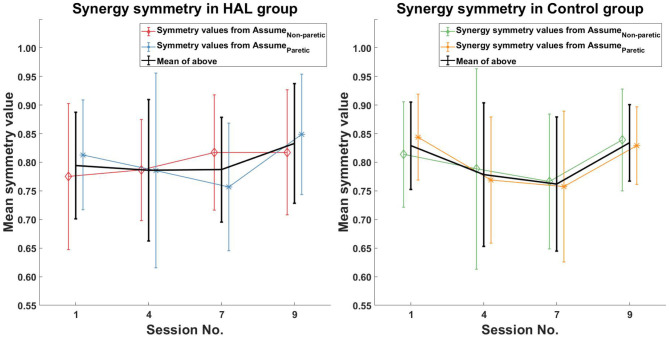
Muscle synergy symmetry comparison. No initial change in muscle synergy symmetry was observed in the HAL group, with an observed increase in symmetry values in the last session. For the Control group, a decrease in muscle synergy symmetry was observed initially, followed by an increase, which brings it back to pre-therapy levels.

Comparison of muscle synergy modules between all sessions (1st session against 4th, 7th, and 9th sessions) did not reveal any significant differences between session in both the HAL group and Control group (Figure 6 Left and Right).

**Figure 7:**
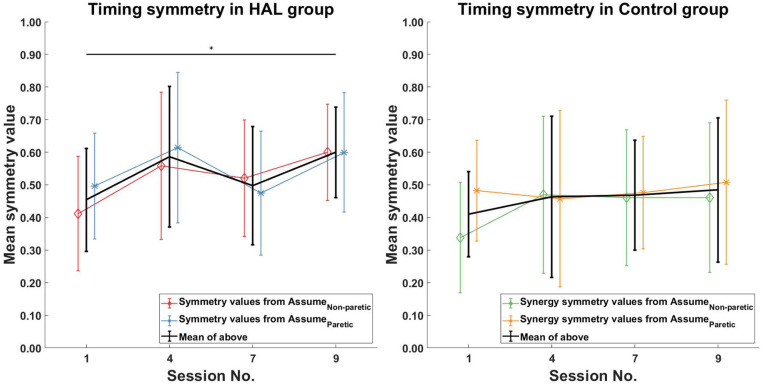
Timing symmetry comparison. Significant differences in timing coefficients were observed between the 1st and 9th session in the HAL group (Left). However, no significant differences in timing coefficients were observed for the Control group. Black asterisks denote significant increases in symmetry when comparing the 1st session to all other sessions. Lines with symbols denote the mean, while errorbars denote standard deviations.

For the symmetry in the corresponding timing coefficients of the matched synergies, increasing symmetry was only observed in the HAL group (Figure 7 Left) between the 1st and 9th session [0.45 ± 0.16 -> 0.6 ± 0.14 (*p* = 0.0391*CI* = [−0.2746, −0.0002])]. However, no significant differences in timing symmetry was observed in the Control group (Figure 7 Right).

**Figure 8:**
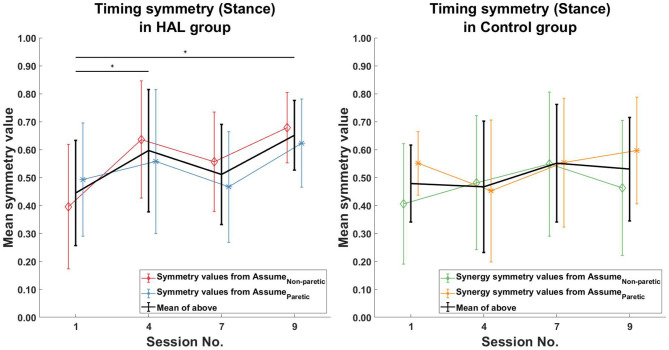
Timing symmetry comparison in stance phase. Significant differences in timing coefficients were observed between the 1st session, with the 4th and 9th session in the HAL group (Left). However, no significant differences were observed in the Control group. Black asterisks denote significant increases in symmetry when comparing the 1st session to all other sessions. Lines with symbols denote the mean, while errorbars denote standard deviations.

When comparing only the timing symmetry during the stance phase, a significant increase can be observed for the HAL group (Figure 8 Left) between the 1st and 4th session [0.44 ± 0.19 -> 0.60 ± 0.22 (*p* = 0.0391*CI* = [−0.2803, −0.0178])], 1st and 9th session [0.44 ± 0.19 -> 0.65 ± 0.12 (*p* = 0.0039*CI* = [−0.3111, −0.0923])]. However, no significant differences in timing symmetry during stance phase was observed in the Control group. (Figure 8 Right).

**Figure 9:**
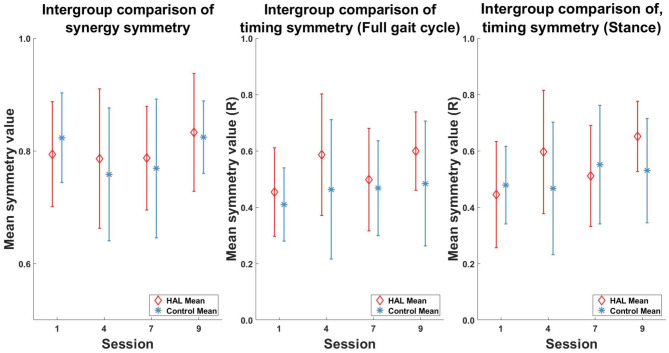
Intergroup comparison of muscle synergy and timing, pre- and post-therapy. No significant differences in muscle synergy symmetry and timing symmetry was observed between groups in all the sessions.

Comparisons between the two patient groups (HAL and Control) did not show any significant differences in muscle synergy symmetry (Figure 9 Left) and timing symmetry (Figure 9 Center and Right).

### 3.7. Verification of Between Sensor Detection

**Figure 10:**
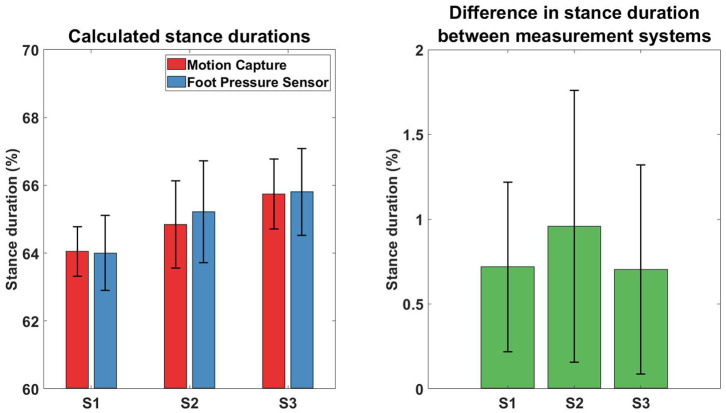
Comparison of calculated stance duration between different measurement systems. Results of the stance duration from three subjects, measured with different gait tracking systems. Left plot depicts the mean and standard deviation of the values recorded from the two measurement systems, while the Right plot depicts the difference between the values from both systems.

Figure 10 depicts the mean and standard deviation stance duration values of the 3 healthy subjects, from both measurement systems. Stance duration values were similar (Figure 10 Left) between both systems and the differences (Figure 10 Right) were within 2%.

### 3.8. Excluded Patients

Two patients from the HAL group (R5, and R9) were excluded because their FAC was evaluated to be 3 during the first session. Their results are presented individually below in Table 4.

**Table 4:** Results of excluded patients.

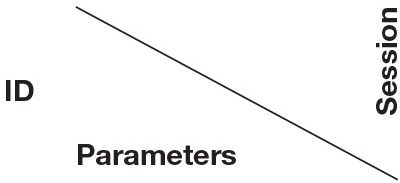	**1st**	**4th**	**7th**	**9th**
R5	FIM-Loco	2	–	–	7
	FIM-Motor	78	–	–	90
	FMA-LE	20	–	–	27
	Muscle synergy symmetry	0.7847	0.9184	0.8693	0.9043
	Timing symmetry (full gait cycle)	0.4675	0.6846	0.5643	0.7361
	Timing symmetry (stance)	0.5462	0.8110	0.7749	0.8013
	Stance time ratio	0.8647	0.9574	0.9406	0.9101
	Number of synergies (paretic)	3	2	3	3
	Number of synergies (non-paretic)	2	3	3	4
	Stance percentage (%) (paretic)	74.53	69.33	64.91	64.66
	Stance percentage (%) (non-paretic)	74.35	72.68	69.15	70.23
R9	FIM-Loco	2	–	–	5
	FIM-Motor	68	–	–	82
	FMA-LE	29	–	–	30
	Muscle synergy symmetry	0.9444	0.9632	0.9063	0.9544
	Timing symmetry (full gait cycle)	0.9145	0.8172	0.8277	0.8788
	Timing symmetry (stance)	0.9190	0.8022	0.8453	0.8375
	Stance time ratio	0.9806	0.9843	1.0272	0.9667
	Number of synergies (paretic)	3	3	3	3
	Number of synergies (non-paretic)	3	3	3	3
	Stance percentage (%) (paretic)	73.28	71.26	72.56	68.36
	Stance percentage (%) (non-paretic)	73.93	72.42	71.90	70.52

*This table lists the results of patients who were excluded from analysis due to their FAC improving to 3, between recruitment and the 1st therapy session*.

Both patients were evaluated to have higher motor function scores (FIM-Loco, FIM-Motor, FMA-LE) after the course of therapy.

In terms of muscle synergy symmetry, R5 was around the group average symmetry (0.78 against 0.79), while R9 was above it (0.944 against 0.79) at the start of the therapy program (Figure 6 Left). Only R5 had a change in muscle synergy symmetry over the course of therapy, while for R9, it remained stable. Both patients were above the group average symmetry value at the end of therapy [0.90 (R5) and 0.95 (R9) against 0.83].

Timing symmetry of the full gait cycle for R5 were around the group average at the 1st session (0.47 compared to 0.45 ± 0.16) (Figure 7 Left). Changes in timing symmetry was observed to fluctuate over the course of therapy, but the general trend points to an increase in timing symmetry (from 0.47 to 0.74, R5, Table 4). The timing symmetry value at the 9th session was at the edge of the group average (0.74 compared to 0.6 ± 0.14). A similar trend of increase in the timing symmetry during the stance was also observed for R5 (from 0.55 to 0.80, R5, Table 4). However, in this case, timing symmetry during stance was within the group average at the 1st session (0.55 compared to 0.44 ± 0.19), but it was above the group average at the 9th session (0.80 compared to 0.65 ± 0.12) (Figure 8 Left).

For patient R9, timing symmetry of the full gait cycle was above the group average at both 1st and 9th sessions [0.91 as compared to 0.45 ± 0.16 (1st)] and [0.88 as compared to 0.6 ± 0.14 (1st)] (Table 4 and Figure 7 Left). The general trend observed is a slight decrease in timing symmetry. In the case of timing symmetry during stance phase, the pattern and trend holds (i.e., R9's timing symmetry during stance above group average, but showing a slight decrease) (1st: 0.91 compared to 0.45 ± 0.16) (9th: 0.84 compared to 0.65 ± 0.12) (Table 4 and Figure 8 Left).

Stance time ratios of both patients were similar to the group average (0.86 and 0.98 as compared to 0.93 ± 0.18) (Figure 4 Right) at the 1st session, however there were differences in trends between patients. Stance time ratio for R5 was observed to decrease, while in R9, only minor fluctuations were observed. Stance time ratios of R5 and R9 at the 9th session were within group average (0.91 and 0.97 against 0.94 ± 0.11) (Figure 4 Right).

At the 1st session, stance percentages for both limbs of both subjects were within the group averages (Paretic: 68.9 ± 9%, Non-paretic: 78.5 ± 9) (Figure 4 Left). Stance percentages were observed to steadily decrease in R5, while in R9, values tend to fluctuate. Similar to the 1st session, the stance percentages for both limbs were within the group average at 9th session, with a net decrease in stance percentages.

## 4. Discussions and Conclusions

Our study aims to quantify gait symmetry changes with muscle synergies and evaluate differences in muscle coordination between patients undergoing robotic gait training and conventional gait training (HAL group vs. Control group). Our results showed that this method is a good complement to clinical scores and reveal some key differences between patients in different groups.

### 4.1. Comparison With Multiple Number of Synergies

Muscle synergies and their corresponding timings were compared using multiple number of synergies extracted from different conditions (section 2.5.5). The key reason behind this comparison is to allow direct comparison between the paretic and non-paretic limbs, which typically have different number of synergies (Clark et al., 2010). However, imposing the same number of synergies on both the paretic and non-paretic limb would make estimation of the contents of muscle synergies difficult, since either too many or too few synergies were used. Our method attempts to resolve this by taking the mean of multiple comparisons with different number of synergies. The results obtained from such comparisons (Figures 6, 7) allowed us to quantify the trend in muscle coordination change through in-patient rehabilitation. We believe that quantifying trends in muscle usage symmetry would provide a way to quantify trends in recovery, thereby facilitating the transfer of this analysis method to the clinical domain. While there is a possibility that each measurement condition might either overestimate or underestimate the number of synergies for the individual limbs, taking the average from each measurement condition would reduce the impact of overestimation or underestimation. Furthermore, since patients were only compared with themselves, they were their own control, which also helps to reduce estimation errors.

### 4.2. Number of Muscle Synergies and Symmetrical Control

The number of muscle synergies that can be extracted was suggested to be an indication of the motor complexity in patients, with a higher number of synergies correlating to better control of the limb (Clark et al., 2010; Cheung et al., 2012). This would suggest that more patients in the HAL group had better motor complexity after therapy, as compared to the Control group (Paretic Limb column, Table 3). However, relating the number of synergies to motor complexity would not account for some cases, where patients had a reduction in the number of synergies (R10, Paretic Limb column, Table 3). Cheung et al. (2012) noted that merging (decrease in muscle synergy) and fractionation (increase in muscle synergy) can occur in stroke patients as a response to cortical damage. For example, R10 was shown to do quite well in clinical evaluation tests (Table 2). Hence, a decrease in the number of synergies does not necessarily indicate patients get worse. Although the general trend indicates having more number of synergies would be better (Clark et al., 2010), there might be other factors that can contribute to the change in the number of muscle synergies. In another related work, Hashiguchi et al. (2016) noted that muscle synergies in the lower limbs of patients can exhibit both merging and fractionation over the course of therapy.

Instead of examining whether patients increase or decrease their number of muscle synergies, we would like to point out that the number of synergies could possibly be related to gait symmetry. With the naive assumption that when both limbs have the same number of synergies, muscle activation in both limbs are symmetrical, it is suggested that more patients in the HAL group had better symmetry after therapy, as compared to those in the Control group (Table 3). However, what is interesting to note that R10 decrease the number of synergies in the paretic limb to match the number in the non-paretic limb after the course of therapy (9th session, Table 3). This is suggested in Madhavan et al. (2010) where the brain tries to balance control such that both limbs would have the same level of control.

### 4.3. Lack of Correlation Between Clinical Scores and Muscle Synergy Symmetry

In our study, a lack of correlation between stance symmetry (stance time ratio, Figure 4 Right) and scores from clinical evaluation tests (FIM and FMA scores, Table 2) was observed, as was also noted in a previous study (Patterson et al., 2010a). There was significant improvement in clinical scores of patients in the both groups, however, this improvement does not seem to be reflected in the improvement of the stance time ratio. This could be because the FIM and FMA evaluations were meant to evaluate patients in terms of ability in daily living and gross neurological health, not in terms of specific gait parameters.

### 4.4. Effects of Therapy Type on Stance Percent Symmetry and Stance Time Ratio

Our results in stance percentage comparison suggest that the Control group were less asymmetric after conventional therapy, however, the large standard deviation in stance duration in the 9th session (Figure 4 Left) could indicate variable individual differences in recovery. In contrast, although the HAL group was still asymmetric after robotic therapy, the standard deviation of stance percentage for both limbs were small, which could be due to the support from the robotic exoskeleton used during training. From a stance time ratio perspective, both groups did not improve their gait symmetry over the course of their respective therapies, but mean values of stance time for the HAL group could be said to be sufficiently high ranging from 0.93 to 0.94, while the Control group stance time mean values were ranging from 0.83 to 0.87. The lack of change in stance time ratio could be that the patients might already be “symmetric” enough, given that the stance time ratios were close to the perfect symmetry of 1.

Our results seem to be similar to the results of Patterson et al. (2015), found that patients did not significantly improve spatiotemporal gait symmetry over a course of conventional therapy. However, this result disagrees with results from an earlier study by Routson et al. (2013). In the earlier study (Routson et al., 2013), it was found that body weight support and manual training, combined with overground walking, was able to improve gait symmetry over a course of therapy. This suggests that gait symmetry could be highly dependent on the type of therapy the patients are receiving. Patterson et al. (2015) did point out their study was retrospective and one of the limitations in their study was that the detailed records of the treatment was not available. Since it was suggested that certain therapy methods could help patients regain gait symmetry (Routson et al., 2013), one future consideration could be to determine the factors contributing to the improvement of gait symmetry and translate these factors into control paradigms for robotic exoskeletons.

### 4.5. Improvement in Temporal Muscle Coordination in the HAL Group

The improvement of synergy timing symmetry shown in the HAL group may characterize the effect of HAL sessions on the neural gait control, in comparison with conventional gait training. Routson et al. (2013) showed that both timing and composition of some of the synergy modules became closer to healthy group after a treadmill based gait training in stroke patients. In this regard, HAL's effect of gait improvement may resort more to alterations of activation timings rather than the composition of synergies, compared to conventional gait training.

The activation of muscle synergies was suggested to be cortically-controlled, based on results from primate studies with implanted electrodes in the brain and upper limbs of monkeys (Overduin et al., 2015). Studies of reaching humans in stroke patients also support this notion, where it was observed that muscle synergy compositions of stroke patients were consistent with healthy subjects (Cheung et al., 2009). Improvement of timing symmetry observed in our HAL group may be considered as an indication of improvement of cortical control of gait after the HAL sessions, which was not observed in the control group. In fact, Routson et al. (2014) showed that spontaneous adaptability of synergy timing is limited during gait of stroke patients in comparison to healthy controls. Hence, HAL's ability to assist cortical function in control of synergy activation was considered. In contrast, Gizzi et al. (2011) showed that the synergy modules are altered but not the synergy activation timings in stroke patients. However, the main difference is that the group of patients analyzed in Gizzi et al. were late sub-acute phase patients (8–20 weeks after stroke onset), while the patients in our study were in the early sub-acute phase (2–4 weeks after stroke onset). The difference in stroke onset duration might contribute to a difference in results. Definition of the phases of stroke were based on the latest consensus defined in Bernhardt et al. (2017). Early training with a course of HAL can help achieve earlier recovery of synergy timings which could otherwise occur later, as this recovery is not observed in the Control group. A methodological difference should be noted here; while Gizzi et al. (2011) did their comparison between groups, we first compared synergy timings within each individual patient, then compared all the obtained symmetry indices between groups.

### 4.6. Relation Between Muscle Coordination and Stance Symmetry

Another point of note is that despite muscle synergy and timing symmetry improved significantly, stance time ratios are relatively unchanged after the course of therapy. This was observed for both groups of patients (Figure 4 Right). This is interesting because if patients were able to improve symmetrical muscle coordination, improvement in stance ratio symmetry would be expected. We hypothesize that the stance time symmetry would be related to timing symmetry of the muscle synergies during stance phase and analyzed timing symmetry during stance phase for the patient groups. However, results showed the opposite, where timing symmetry during the stance phase improved consistently in the HAL group (Figure 8 Left), but stance time ratios were relatively unchanged (Figure 4 Right, HAL group). This observation should be studied further to clarify the relations between stance ratio symmetry and muscle synergy timing symmetry. Perhaps study with a longer duration could examine in greater detail how gait symmetry changes as the patients progresses from subacute therapy to chronic therapy.

### 4.7. Muscle Usage and Body Weight Bearing on Limbs

Patterson et al. (2015) proposed that improvement in swing symmetry could be correlated with increased body weight bearing on the paretic limb. Further support for this correlation comes from a study by Hendrickson et al. (2014). They found a correlation between balance in quiet standing and gait, that is, patients that walked asymmetrically had similar patterns of asymmetry during balance. Similarly, Yavuzer et al. (2006) found that balance training that compelled patients to bear more weight on their paretic side also improved gait symmetry. In such a context, we expect improvements in the symmetry of muscle synergy activations (i.e., timing symmetry) to increase during the stance phase. We think it could be that paretic limb weight loading was facilitated by the HAL exoskeleton during gait training, as the exoskeleton compensates for weakness in the paretic limb by providing compensatory torque around the knee and hip joints during walking in post-stroke patients. Although body weight loading on the paretic leg was not measured in our study, it is hypothesized that the symmetrical activation of muscles are correlated with symmetrical body weight loading in both limbs. Hence, if the muscle coordination in the paretic limb is similar to the non-paretic limb, then increased usage of the paretic leg is assumed. The increased symmetry of muscle timing coordination in the HAL group (Figures 7, 8 Left) appears to support this hypothesis. The lack of symmetry improvement in muscle coordination in the Control group (Figures 7, 8 Right) gives further support to this hypothesis. There were indications that the amount load the limb bears would change EMG activity from a study with varying body weight support (Ivanenko et al., 2002). Measuring ground contact forces over the course of therapy could be a good future consideration.

### 4.8. Limitations of Study

One limitation of this study is the number of muscles analyzed was small (six muscles per limb). However, the six muscles chosen were major muscle groups contributing to lower limb movement in the sagittal plane, which would be sufficient as part of the clinical evaluation process.

The other limitation could be that the Control group was recruited from hospitals that do not have access to motion tracking facilities, hence the use foot pressure sensors. There might be differences in tracking accuracy in the data collected. However, a small verification test comparing the data captured with motion tracking and the foot pressure sensor showed that the accuracy did not differ much (about 2% difference, Figure 10). Hence, the use of different methods of tracking stance duration would not affect our results much. However, future considerations should include capturing spatiotemporal gait parameters using the same type of sensors, for example, using wearable technology to expand data capture in community hospitals interested in participating in such studies.

Another limitation could be that stance percentage and stance time ratio of patients during recruitment were not considered. In general, the Control group were more asymmetric, in terms of stance time ratio, as compared to the HAL group (Figure 4 Right), and were having a higher stance percentage, as compared to the HAL group (Figure 4 Left). This difference may cause differences in the rate of recovery between groups.

A final limitation could be that the exact details of the exercises performed by the patients during conventional regular physiotherapy sessions were not tracked. Tracking every exercise for each patient requires tremendous effort by each individual therapist and therapy center, which is currently difficult to implement. Future studies should consider designing tools to ease data entry.

## 5. Conclusions

In conclusion, one main contribution of this study is that muscle synergy analysis is able to differentiate between patients undergoing different types of therapy, in terms of gait symmetry. However, clinical scores were unable to do so. This is an important result because functional clinical tests manually evaluate abilities in daily living, not the neurological state of patients. From our results, robotic therapy appear to be able to help patients regain temporal symmetry (muscle synergy timings) over a period of 3 weeks. However, the lack of kinematics, coupled with the high variability of recovery in the Control group contributed to mixed results. Muscle coordination symmetry appear to be quantifying a different aspect of gait symmetry, as compared to spatiotemporal measures, however, this is still unclear and future works should consider clarifying the differences and underlying mechanisms influencing gait symmetry to provide targeted therapies.

## Data Availability Statement

The datasets generated for this study are available on request to the corresponding author.

## Ethics Statement

This study was carried out in accordance with the recommendations of the University Guidelines for Clinical Trials, Institutional Review Board of University of Tsukuba Hospital, with written informed consent from all subjects. All subjects gave written informed consent in accordance with the Declaration of Helsinki. The protocol was approved by the Institutional Review Board of University of Tsukuba Hospital.

## Author Contributions

CT and HK collected, analyzed and interpreted the data, wrote and drafted the manuscript. HW, AMar, and AMat planned and administered HAL treatment. AMar diagnosed the patients and prescribed HAL treatment. YH and MY provided the important comments for the clinical part of the study and helped developing HAL treatment. YS originally developed the robot suit HAL and conceived the idea of HAL treatment. KS designed the analysis and provided essential insight for the paper. All authors made critical revisions of the manuscript and approved the final version.

## Conflict of Interest

YS is the CEO, shareholder, and director of CYBERDYNE Inc. which produces the robot suit HAL. CYBERDYNE was not involved in study design, data collection, analysis, writing, or submission of this article. The remaining authors declare that the research was conducted in the absence of any commercial or financial relationships that could be construed as a potential conflict of interest.
